# Cutaneous secondary localization of multiple myeloma at Heparin injection sites

**DOI:** 10.1002/ski2.153

**Published:** 2023-02-02

**Authors:** Emma Borg, Louise Muguet Guenot, Antoine Tichadou, Mathias Colantonio, Emilie Baubion

**Affiliations:** ^1^ Dermatology Department CHU Martinique Fort‐De‐France France; ^2^ Hematology Department CHU Marseille Marseille France; ^3^ Pathology Department CHU Martinique Fort‐De‐France France

## Abstract

We report two cases of multiple myeloma skin localizations at Heparin injections sites in patients followed at the University Hospital of Martinique. These skin manifestations on traumatized areas are a marker of aggressiveness in the natural history of multiple myeloma.

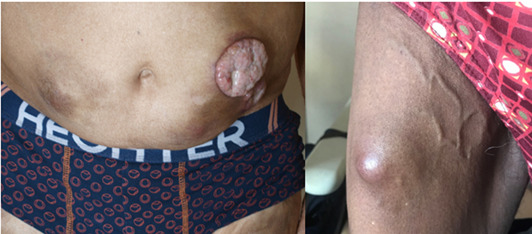

## ETHICS STATEMENT

Not applicable.

1

**FIGURE 1:**
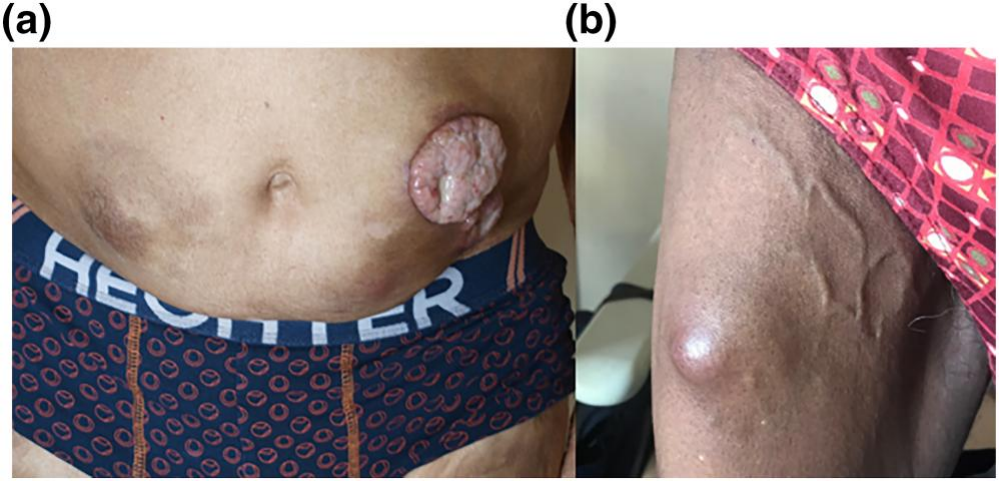
Clinical presentation of patient 1 (a) and 2 (b)

Dear Editor,

Multiple myeloma (MM) is a malignant proliferation of plasmocytic cells producing in excess a monoclonal immunoglobulin. As its incidence grows, clinicians must recognize the disease at the earliest stage. Cutaneous secondary localizations of MM can occur far from bone lesions or even without any bone localization.^1^ A 77‐year‐old man was diagnosed with indolent MM in 2011. His IgA lambda spike was of fortuitous discovery, with plasmocytic medullar infiltration of 38% and no end‐organ damage at diagnosis. He underwent three therapeutic lines after progression: MPV (melphalan, prednisone, bortezomib), VRD (bortezomib, revlimid, dexamethasone), PED (pomalidomide, endoxan, dexamethasone) since 2018. In 2019, he presented subcutaneous nodules on the abdomen and thighs at Heparin therapy injection sites (Figure 1a). Lesions were painless, sized from 6 to 8 cm. Multiple myeloma was relapsing with multiple bone localizations. The cutaneous biopsy showed a monoclonal plasmocytic infiltration and immunohistochemistry positivity for CD138 and IgA lambda secreting population. Palliative care was proposed. The second patient was 81 years‐old (WHO Performance Status 1 with kidney insufficiency), diagnosed with MM ISS‐R stage II (intermediate prognosis) in 2017. He had an IgA lambda spike (5.6 g/L) and a plasmocytic medullar infiltration of 58% at diagnosis. Moreover, he had hypercalcaemia, anaemia, renal failure and bone lesions. He underwent two therapeutic lines: MPV (melphalan, prednisone, bortezomib) and RD (revlimid, dexamethasone). In 2018, he was treated by Heparin after a venous thrombosis. One month later, cutaneous nodules appeared on Heparin injection sites (Figure 1b). Skin biopsy revealed a secondary localization of MM. He received a third therapeutic line with PED (pomalidomide, endoxan, dexamethasone) and radiotherapy was proposed. He died 1 month later.

The poor prognosis of extramedullary cutaneous localization of MM is consensual. Jurczyszyn et al.^2^ reported 53 cases of skin secondary MM localizations, in a multi‐institutional retrospective study on 23 centers in Europe and America. Lesions were red‐violets nodules or lumps, sometimes ulcerated, commonly on the chest, lower extremity, back and buttocks. One patient had lesions on an abdomen area of repeated subcutaneous injection, another on a scare zone of amputation. Skin localizations appeared at any times of the disease, after a median of three therapeutic lines, with a median of 2.2 years after diagnosis. The overall survival was 8.5 months. Plasmablastic (aggressive) myeloma was found in 60% of cases.

Avivi et al.^3^ reported 127 patients with extramedullary relapse of MM in a retrospective study in 15 centers between 2010 and 2018. Twenty‐nine percentage had soft tissue and skin involved sites.

Moreover, one cutaneous localization after pacemaker implantation^4^ and two secondary localizations occurring on the tract of central venous catheters^5^ have been reported. They were mainly nodular forms, such as our patients.

The physiopathology of extramedullary MM is poorly known. Marchica et al.,^6^ suggested the role of pro‐inflammatory cytokines secreted by cutaneous cells (CCL27) binding on CCR10 receptor overexpressed on malignant plasmocytes. The low rate of bone marrow adhesion factors such as CXCR4 could also explain extramedullary bone localizations. Geng et al.^7^ suggested a chemokine auto‐secretion of CXCL12 (CXCR4 ligand for bone marrow retention) by circulating plasmacytoma cells, getting them independent from bone homing. In a recent article, Sevcikova et al.^8^ summarize the current knowledge on underlying mechanisms leading to extra‐medullary localizations; most of them are based on couples of chemokines and their receptors with a focus of attention on bone marrow environment.

These two reports of secondary cutaneous lesions of MM occurring at Heparin injection sites, comparable to extramedullary MM lesions on vascular abords, scars, trauma and surgery sites, could be linked to the same behaviour of plasmocytic cells, explaining this koebnerisation. Thus, it is a manifestation of aggressive MM.

## AUTHOR CONTRIBUTION

**Emma Borg**: Writing – original draft (Lead). **Louise Muguet Guenot**: Writing – review & editing (Lead). **Mathias Colantonio**: Investigation (Equal). **Antoine Tichadou**: Validation (Equal). **Emilie Baubion**: Supervision (Supporting).

## CONFLICTS OF INTEREST

The author declares that there is no conflict of interest that could be perceived as prejudicing the impartiality of the research reported.

## FUNDING INFORMATION

This research received no specific grant from any funding agency in the public, commercial, or not‐for‐profit sectors.

## Data Availability

Data sharing is not applicable to this article as no new data were created or analyzed in this study.
